# Development of Aptamer-Based TID Assays Using Thermophoresis and Microarrays

**DOI:** 10.3390/bios9040124

**Published:** 2019-10-14

**Authors:** Tracy Kurth, Sandra Witt, Svenja Bolten, Janice-Joy Waniek, Carlotta Kortmann, Antonina Lavrentieva, Thomas Scheper, Johanna-Gabriela Walter

**Affiliations:** Institute of Technical Chemistry, Leibniz University of Hannover, Callinstraße 5, 30167 Hannover, Germany; kurth@iftc.uni-hannover.de (T.K.); kuhlmeier@iftc.uni-hannover.de (S.W.); bolten@iftc.uni-hannover.de (S.B.); waniek.janice@yahoo.com (J.-J.W.); kortmann@iftc.uni-hannover.de (C.K.); lavrentieva@iftc.uni-hannover.de (A.L.); scheper@iftc.uni-hannover.de (T.S.)

**Keywords:** Aptamer, microarray, protein detection, target-induced dissociation (TID), biosensor, thermophoresis, vascular endothelial growth factor (VEGF)

## Abstract

Aptamers are single-stranded oligonucleotides which can be used as alternative recognition elements for protein detection, because aptamers bind their targets with a high affinity similar to antibodies. Due to the target-induced conformational changes of aptamers, these oligonucleotides can be applied in various biosensing platforms. In this work, aptamers directed against the vascular endothelial growth factor (VEGF) were used as a model system. VEGF plays a key role in physiological angiogenesis and vasculogenesis. Furthermore, VEGF is involved in the development and growth of cancer and other diseases like age-related macular degeneration, rheumatoid arthritis, diabetes mellitus, and neurodegenerative disorders. Detecting the protein biomarker VEGF is therefore of great importance for medical research and diagnostics. In this research, VEGF-binding aptamers were investigated for the systematic development of a target-induced dissociation (TID) assay utilizing thermophoresis and microarrays. The established aptamer-microarray allowed for the detection of 0.1 nM of VEGF. Furthermore, the systematic development of the TID method using the VEGF model protein could help to develop further TID assays for the detection of various protein biomarkers.

## 1. Introduction

Protein detection is of great importance for medical research and diagnostic approaches. Until now, only antibody-based protein analysis has been available. Complementary to antibodies, aptamers could become advantageous recognition elements for protein analytics.

Aptamers are synthetically produced, single-stranded oligonucleotides consisting of deoxyribonucleic acid (DNA) or ribonucleic acid (RNA) and can be seen as an alternative to antibodies [1]. These oligonucleotides are capable of binding proteins [2], peptides [3], small molecules [4] or even whole cells [5] with high affinity in the micro-to-picomolar range [1]. The secondary and tertiary structures of aptamers, including hairpin loops, pseudo knots, and guanine (G)-quadruplexes, are of great importance for the formation of a three-dimensional (3D) structure and, thus, for target-binding [6]. The major inter- and intra-molecular interactions of aptamers are based on hydrogen bonding, electrostatic interactions, and hydrophobic interactions [6]. The 3D structure of an aptamer depends on the temperature, pH value, and ionic strength of the solution as well as on the sequence itself [6] and can be determined by using nuclear magnetic resonance (NMR) spectroscopy or x-ray crystallography [7]. Aptamers are selected against a specific target by a process termed the Systematic Evolution of Ligands by EXponential enrichment (SELEX) [7]. During the SELEX process, chemically synthesized oligonucleotide libraries with randomized sequences are used and high affinity sequences are selected after binding the target [7]. Furthermore, the specificity of aptamers can be high, since some of these oligonucleotides can even differ between enantiomers [6]. Because of their high binding affinity and specificity, aptamers are applied in several technologies including sensor platforms [4], affinity chromatography [6,8,9], and microarrays [10,11]. One of the advantages of aptamers in comparison to antibodies is their cost-efficient production by chemical synthesis including their easy and directed modification with fluorophore, linker, quencher, or other functional groups [11]. Moreover, aptamers can easily be immobilized on surfaces (e.g., microarrays), are stable at high temperatures, and do not denature as fast as proteins [11]. In addition, immobilized aptamers are stable and can be stored for a long period of time [11]. Since the target-binding of the aptamer is reversible, regeneration and multiple usage is possible [12,13,14,15]. 

The mentioned advantages of aptamers and sophisticated synthesis techniques make ready-to-use aptamer-microarrays a promising alternative to protein-microarrays [11]. Printing technologies like fluid droplet dispensing allow for the high throughput generation of microarrays and photochemical methods like photochemical patterning or photolithography—which can also be combined with the in-situ synthesis of capture oligonucleotides—can be applied [16]. The development of aptamer-microarrays requires the careful consideration and experimental investigation of suitable buffers, spacers, immobilization techniques, microarrays surfaces, etc. [11,15,17]. Currently, a few commercial aptamer-microarray products are available for the detection of biomarkers by SomaLogic [11]. Moreover, despite the current limitations in the application of aptamer microarrays, they represent a suitable platform technology to develop and optimize aptamer-based assays [11].

Concerning the detection of the binding event, several principles can be used for aptamer microarrays. As in antibody microarrays, simple binding assays can be performed in the forward phase microarray format, which requires the labeling of the analyte, which is not easy to realize, especially when using complex samples. To avoid the labeling of the analyte, aptamer microarrays can also be set up in a sandwich format [11]. In this sandwich format, one aptamer is immobilized on the microarray, and the bound target is detected by a second, labeled aptamer. These sandwich assays require the availability of two aptamers that are directed against different “aptatopes” of the target. In contrast, other aptamer-based assay principles are aptamer specific and cannot be realized using antibodies [11]. These assays include the target-induced reassembly of aptamer fragments and the target-induced dissociation of complementary oligonucleotides [4].

In this work, vascular endothelial growth factor (VEGF) was used as a model analyte. VEGF can take part in the development of different diseases like diabetes mellitus [18,19], age-related macular degeneration [20], rheumatoid arthritis [21,22], and neurodegenerative diseases [23] like Parkinson’s disease, Alzheimer’s disease, and amyotrophic lateral sclerosis. In 2004, the first aptamer approved by the Food and Drug Administration (FDA) was a VEGF-targeting aptamer called pegaptanib [20,24]. Since then, different research groups have selected various aptamers against VEGF, such as the aptamers named V7t1 and Del5-1 [2,25,26,27]. The V7t1 aptamer contains a G-quadruplex structure [13]. G-quadruplexes comprise G-rich regions which form more than one quadratic shape of the four guanines named G-quartet which are stapled vertically over each other [28]. Moreover, various G-quadruplex structures are known to target different proteins like VEGF [13], thrombin [29], and nucleolin [30]. Therefore, G-quadruplexes can be considered promising target binding sites in aptamers that can be useful for designing detection strategies based on the target-induced structural changes of the aptamer [29].

Since most aptamers undergo structural changes upon target-binding, their target-induced conformational reorganization can be utilized for detection strategies, e.g., in aptamer-microarrays [31] or other biosensing platforms [21]. The nucleic acid-based sequence of aptamers allows for the design of oligonucleotides which are complementary to the target-binding site of the aptamer and can compete with the target in various assays [31,32]. The target-induced dissociation (TID) of complementary oligonucleotides allows for the detection of targets without labeling the analyte [32]. The aptamer-based TID mechanism has been proven to allow for highly sensitive detection in several applications including lateral flow assays [33], quantitative polymerase chain reaction (qPCR) [34,35], and other biosensing platforms [19,21]. The TID mode thereby uses oligonucleotides complementary to the aptamers sequence, which form a duplex with the aptamer via hybridization [4]. In the presence of the aptamer target molecule, the complementary oligonucleotide dissociates from the aptamer while the aptamer forms a complex with the target [4]. This dissociation can be optically detected by modifying the aptamer and/or the complementary oligonucleotide with fluorophores or other labels [4].

Until now, TID mechanisms have been mainly applied to small molecule detection [31,34,35]. Nonetheless, since TID assays do not require the labeling of the analyte (as required in conventional competitive assays) or the availability of two specific ligands binding to different binding sites of the analyte (as required for sandwich assays), the transfer of the TID mechanism to the detection of proteins could be advantageous. Therefore, efforts for TID-based protein detection have been already performed. Freeman et al. developed an optical aptasensor for VEGF detection using the TID method based on fluorescence resonance energy transfer with a detection limit of 1 nM [19]. The VEGF-binding aptamer was used for the experiment, and the G-quadruplex of the aptamer was exploited for the detection [19]. Therefore, an oligonucleotide complementary to the G-rich region of the aptamer was used to suppress quadruplex formation in absence of VEGF [19].

In our research, we focused on the schematic development of a TID assay via thermophoresis and an aptamer-microarray. Using microarrays with immobilized aptamers excludes costly and unstable antibodies for VEGF detection and sustains the reusability of the aptamer. Therefore, we first investigated the affinity and specificity of different VEGF-binding aptamers by thermophoresis. Thereafter, an oligonucleotide complementary to the target-binding site of the chosen aptamer was designed for setting up a TID assay. Finally, the TID mechanism was transferred onto a microarray surface to prove the applicability of TID-based protein detection in the aptamer-microarray format. This systematic development of a TID assay for detecting the VEGF model protein can be transferred to various biosensing platforms for the detection of different biomarkers with other aptamers.

## 2. Materials and Methods

### 2.1. Chemicals

NaCl and EDTA (ethylenediaminetetraacetic acid) were purchased from Thermo Fisher Scientific, Waltham, Massachusetts, USA. Na_2_HPO_4_, KCl and KH_2_PO_4_ were obtained from Sigma-Aldrich, St. Louis, Missouri, USA. Ethanol (99.5%) and Tris were received from Carl Roth GmbH & Co. KG, Karlsruhe, Germany. Tween 20 was purchased from VWR, Radnor, PA, USA. SYBR Green I and II were obtained from Molecular Devices, San José, CA, USA. NaBH_4_ was received from Honeywell Riedel-de Haen AG, Seelze, Germany.

### 2.2. Buffers and Solutions

TBSET (Tris-buffered saline with EDTA and Tween 20) contained 10 mM of Tris, 100 mM of NaCl, 50 mM of KCl, 0.05 mM of EDTA and 0.05% Tween 20 (pH 7). The TBSET without KCl and 0.05% Tween 20 was used for the selection of the aptamers V7t1 and Del5-1, or at least their parent aptamer during the SELEX process [2,36]. Tween 20 was added to the buffer to reduce unspecific hydrophobic interactions. Marušič et al. investigated the increase of the stability of the G-quadruplex from V7t1 by adding potassium ions [13]. Therefore, 50 mM of KCl was added to the buffer. For spotting and immobilizing aptamers onto the microarray (3D-aldehyde glass slides from PolyAn, Berlin, Germany) surface, a spotting solution consisting of 100 mM of NaCl, 50 mM of KCl and 0.05 mM of EDTA (pH 7) was prepared. The preparation of microarray slides required a blocking solution containing 0.1 g of NaBH_4_ dissolved in 10 mL of 99.5% ethanol and 30 mL of PBS (phosphate-buffered saline), which was composed of 137 mM of NaCl, 2.7 mM of KCl, 4.3 mM of Na_2_HPO_4_, and 1.4 mM of KH_2_PO_4 _(pH 7.4). TBSETB (Tris-buffered saline with EDTA, Tween 20 and bovine serum albumin (BSA)) contained TBSET with 1% BSA. All solutions were prepared with deionized water (Arium661, Sartorius AG, Göttingen, Germany).

### 2.3. Oligonucleotides 

Nonaka et al. developed the V7t1 aptamer specifically binding to VEGF with the sequence 5′-TGTGGGGGTGGACGGGCCGGGTAGA-3′ [2]. The spacer sequence attached to the V7t1 aptamer is based on its maternal aptamer, termed Vap7 [2]. During the selection of Vap7, this sequence was part of the primer region in the SELEX process [2]. Lönne et al. investigated the performance of the immobilized V7t1 aptamer using different spacers [8]. The binding affinity of the V7t1 aptamer to VEGF was highest when using the 14 nt spacer based on the primer sequence [8]. The other VEGF-binding aptamer, Del5-1, was developed by Hasegawa et al. [36]. As negative control, the Syl3C aptamer selected by Song et al. against the protein epithelial cell adhesion molecule (EpCAM) was used [37]. The sequences of the used aptamers, as well as their corresponding complementary oligonucleotides and modifications are listed in Table 1. Some oligonucleotides were modified with fluorophores cyanine 5 (Cy5) and fluorescein isothiocyanate (FITC), Dabcyl quencher or a terminal amino group (NH_2_). All oligonucleotides were dissolved in deionized water (Arium661, Sartorius AG, Göttingen, Germany) at a concentration of 100 µM as stock solutions.

### 2.4. Proteins

The human, recombinant protein VEGF-A165 (product ID: GFH44, Cell Guidance Systems Ltd., Cambridge, UK) was produced in *Escherichia coli*. The negative control protein α-chymotrypsin was purchased from ApplyChem GmbH, Darmstadt, Germany. Lysozyme (as an additional negative control) and BSA were purchased from Sigma Aldrich, Germany. All proteins were dissolved in TBSET with a concentration of 15 µM as a stock solution and diluted for the experiments.

### 2.5. Preparation of Microscale Thermophoresis (MST) and Capillary Scan Experiments

Throughout the microscale thermophoresis (MST) experiments, the samples were protected from light including the incubation and the storage time as soon as fluorescent molecules were involved. To ensure that both fluorophores, Cy5 and FITC could be measured in the required device-specific fluorescence range (200–1500 fluorescence counts), a minimum concentration of 25 nM of the FITC-labeled aptamer was needed. To achieve good comparability, this concentration of labeled aptamer was used in all MST experiments.

#### 2.5.1. Characterization of Aptamers via MST

MST experiments were performed to characterize different anti-VEGF aptamers. The advantage of the MST for the determination of K_D_ (dissociation constant) is that neither the aptamer nor the protein has to be immobilized. Therefore, the determination of binding affinities is facilitated without any influence of immobilization. Using this procedure thereby reduces the risk of excluding high affinity aptamers from further investigation, which failed due to non-optimal immobilization in other techniques, such as surface plasmon resonance (SPR) or microarrays. Therefore, the binding affinities towards recombinant human VEGF and the two negative controls lysozyme and α-chymotrypsin were investigated. Furthermore, an aptamer directed against EpCAM (named Syl3C) was used as a negative control. Both aptamers and proteins were dissolved and mixed together in TBSET. The aptamers were fluorescently labeled with FITC or Cy5 (Table 1) and used in a constant concentration of 25 nM, and the protein concentration was varied from 7.5 µM to 3.7 nM (or 0.23 nM) by serial dilution. Before the MST measurements were started, the samples were stored for 20 min at 20 °C in an incubator IPP 30 (Memmert GmbH and Ko.KG, Schwabach, Germany). 

#### 2.5.2. Hybridization and Quenching of Complementary Oligonucleotides via Capillary Scan

The quenching effect of hybridizing fluorescently labeled aptamers with complementary oligonucleotides modified with a quencher was investigated by using the fluorescence scanner of the MST Monolith NT.115. Therefore, the FITC-labeled aptamers were incubated with complementary oligonucleotides modified with a Dabcyl quencher in TBSET. While the aptamer concentration was kept constant at 25 nM, the concentration of the complementary oligonucleotide was varied from 1 µM to 0.49 nM by serial dilution. Before the fluorescence measurement via Monolith was started, the samples were heated for 10 min at 95 °C in a thermoblock VWR 732-1210-Doppio (VWR International GmbH, Darmstadt, Germany) to remove secondary structures in the oligonucleotides. Afterwards, the samples were cooled down to room temperature for 30 min for the hybridization of the complementary oligonucleotide strands.

#### 2.5.3. TID of Complementary Oligonucleotides via Capillary Scan

For the TID experiment via the MST Monolith NT.115, an FITC-labeled aptamer and a complementary oligonucleotide modified with a Dabcyl quencher were used. Both the aptamer and the complementary oligonucleotide were diluted and mixed together in TBSET with a concentration of 50 and 14 nM, respectively. Afterwards, the mixture was heated to 95 °C for 10 min using a thermoblock. Then, the samples were cooled down for 30 min at room temperature for the hybridization of complementary oligonucleotide strands. While the oligonucleotides cooled down, the proteins were diluted in TBSET with a starting concentration of 15 µM. Then, the oligonucleotide solution was mixed with the protein solutions by ratio of 1:1. Consequently, the final concentrations of the aptamer and complementary oligonucleotide were 25 and 7 nM, respectively. The protein concentrations were varied starting from 7.5 µM. Before the MST measurements were started, the mixture of aptamers and oligonucleotides was incubated with the proteins. Therefore, the samples were stored for 20 min at 20 °C in an incubator to keep the temperature constant.

#### 2.5.4. MST and Capillary Scan Settings

After incubation, the samples were filled into standard or premium capillaries (NanoTemper Technology GmbH, Munich, Germany). For samples containing VEGF, premium capillaries with low nonspecific binding (product number: MO-K025) were used to inhibit VEGF from sticking to the glass surface while all other samples were transferred into standard capillaries (product number: MO-K022). After that, the measurements were performed at room temperature with the Monolith NT.115 (NanoTemper Technology GmbH, Munich, Germany) using the NT-Control software Version 2.1.3 (NanoTemper Technology GmbH, Munich, Germany) to obtain capillary scans or analyze the samples by MST. Therefore, the MST power was set to 20%. Depending on the fluorophore, the light emitting diode (LED) power was set to 100% for the detection of FITC-labeled aptamers with the selection of the blue LED. Cy5-labeled aptamers were detected by setting the LED power at 20% and the selection of the red LED. Each experiment was performed three times. For the characterization of the aptamers via MST, the K_D_ model of the NT-Analysis software Version 2.1.3 (NanoTemper Technology GmbH, Munich, Germany) was used to determine the K_D_ values. The hybridization and quenching effects of the complementary oligonucleotides and the TID of complementary oligonucleotides were determined by analyzing the fluorescence data of the capillary scan using the NT-Analysis software Version 2.1.3.

### 2.6. Preparation of Microarray Slides

The slides used for the microarray experiments were 3D-aldehyde glass slides from PolyAn, Germany. The microarray slides were protected from light throughout the whole experiment including the incubation, the washing steps, and the storage time as soon as fluorescent molecules were involved. The following washing and incubation steps were carried out while the microarray slide was shaking on an MTS 4 shaker (IKA Werke, Staufen im Breisgau, Germany) at 300 rpm and at room temperature.

The spotting solution was prepared containing the amino-modified V7t1 aptamer diluted in concentrations of 50, 25, 10, 5 and 1 µM, and the resulting solutions were transferred into a PCR plate. For spotting aptamers onto the microarray slides, the Microarray Spotter NP 2.1 Nanoplotter (GeSiM, Großerkmannsdorf, Germany) was used. Five drops per spot were delivered with a volume of approximately 0.2 nL per drop for each aptamer concentration. The microarray was designed to contain 16 identical blocks. On each of the 16 blocks, every aptamer concentration was spotted in four replicates. Spotting was performed using a Nano-Tip A-J (GeSiM, Großerkmannsdorf, Germany). The amino-modification of the aptamer and the aldehyde group on the microarray surface reacted to an imine. A blocking solution containing NaBH_4_ was used for the reduction of the unstable imine to a stable amine before starting experiments. Therefore, the slides were incubated in a glass chamber with a blocking solution for 10 min.

#### 2.6.1. SYBR Green Staining of Microarray Slides

By incubating the blocked microarray slides with a staining solution containing SYBR Green I and II, the immobilization of the amino-modified aptamer was investigated. SYBR Green I intercalates into double-stranded DNA, while SYBR Green II binds to single-stranded DNA. After incubating the microarray slide in the blocking solution, the microarray slide was transferred into a solution of SYBR Green I and SYBR Green II dissolved in deionized water in a ratio of 1:10,000. The slide was incubated in the SYBR Green staining solution for 5 min. Afterwards, the slide was washed two times with 100 mL deionized water for 1 min. Finally, the microarray slide was dried by compressed air before scanning and analysis.

#### 2.6.2. Hybridization of Complementary Oligonucleotides on the Microarray 

The blocked microarray slide was washed for 1 h with 100 mL TBSET in a glass chamber. Afterwards, the TBSET was removed, and the slide was mounted in a hybridization chamber (NEXTERION IC-16-incubation chamber, Schott, Jena, Germany). The 16 compartments were each loaded with 50 µL of the samples. Therefore, the complementary cV7t1 oligonucleotide labeled with Cy5 was diluted in different concentrations of 0.1, 0.5 and 1 nM in TBSETB and also in TBSETB containing 1 µM of VEGF. Furthermore, the complementary oligonucleotide was diluted in concentration of 1 nM in TBSETB containing 1 µM of α-chymotrypsin, with α-chymotrypsin used as a negative control. Afterwards, 50 µL of each sample were transferred in the wells of the hybridization chamber and incubated for 1 h. In microarray experiments, a longer incubation time was chosen compared to the MST experiments. Using the microarray format with immobilized aptamers results in solid-phase hybridization, which is known to be considerably slower than hybridization in the solution. Here, a high charge density and diffusion effects had to be considered, as they could have resulted in a longer hybridization time [38]. After the incubation, each chamber was washed three times with 50 µL of TBSET for 5 min. Next, the microarray slide was taken out of the hybridization chamber and washed two times in a glass tray filled with 100 mL of TBSET. Then, the slide was dried with compressed air.

#### 2.6.3. Microarray-Based TID Assay for Determination of Sensitivity

To further investigate the developed TID assay and to determine its sensitivity, the microarray slide was washed in TBSET for 1 h after blocking. Then, the microarray slide was fixed in the hybridization chamber. Furthermore, each well was filled with 50 µL of TBSETB and 0.5 nM of the complementary cV7t1 oligonucleotide labeled with the Cy5 fluorophore. The samples were incubated for 1 h. Afterwards, each well was washed three times with 50 µL of TBSET for 5 min. Then the wells were filled with 50 µL of TBSETB and incubated for 10 min. The buffer was removed and wells were incubated with 50 µL of TBSETB and VEGF in different concentrations for 1 h. The concentrations of VEGF prepared in TBSETB were 1000, 100, 50, 10, 5, 1, 0.1 and 0 nM. Afterwards, the wells were washed three times with 50 µL of TBSET for 5 min. Then, the slide was taken out of the hybridization chamber and washed two times in a glass chamber with 100 mL of TBSET for 5 min, and the microarray slide was dried with compressed air.

#### 2.6.4. Scanning and Analyzing Microarray Slides

The microarray slides were scanned using a GenePix 4000B device (Molecular Devices, San José, CA, USA) at 100% laser power and a PhotoMultiplier Tube-Gain (PMT-Gain) between 450 and 650. The wave length was adjusted for each fluorophore. The Cy5 fluorophore was scanned at 635 nm and SYBR Green at 532 nm. Afterwards, the scanned slides were analyzed with the GenePix Pro 7 software (Molecular Devices, San José, CA, USA). The average and the standard deviation of the signal intensity of each concentration were determined.

## 3. Results and Discussion

### 3.1. Comparison and Characterization of Different VEGF-Binding Aptamers via MST

Three VEGF-binding V7t1-Cy5, V7t1-FITC, and Del5-1-Cy5 aptamers and a negative control aptamer (Table 1) were characterized by MST. MST was chosen because it allows for the determination of affinities independent of the possible influences of immobilization, which could reduce affinity in other methods, such as SPR or microarrays.

For the characterization of the aptamers via MST, the fluorescently labeled VEGF-binding aptamers were incubated with the VEGF, lysozyme and α-chymotrypsin proteins. The aptamers were kept at a constant concentration of 25 nM, while the protein concentration was diluted serially from 7.5 µM on.

For the experiments, two V7t1 aptamer variants containing a spacer of 14 nucleotides at the 3′-terminus were used. The first V7t1 variant was additionally labeled with a 3′-terminal Cy5 fluorophore (V7t1-Cy5), while the second variant contained an FITC fluorophore at the 5′-terminus (V7t1-FITC). For future applications with immobilized aptamers, spacers might be helpful to reduce or even prevent steric hindrance. The third VEGF-binding aptamer used for the experiments, named Del5-1, was directly labeled with a Cy5 fluorophore at the 3′-terminus (Del5-1-Cy5).

**Figure 1:**
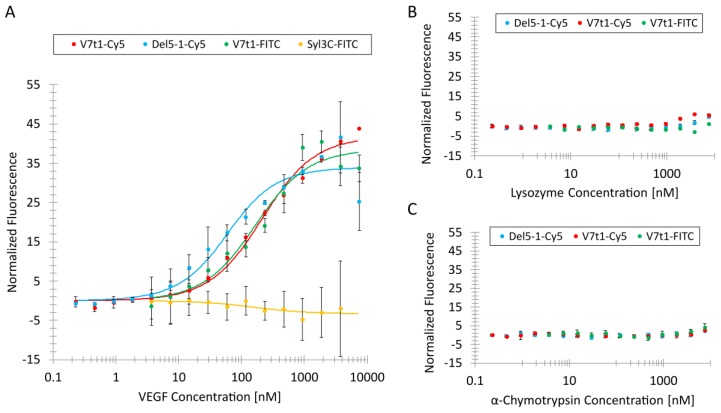
Binding curves for determining the dissociation constants (K_D_) of the different fluorescently labeled aptamers towards (**A**) vascular endothelial growth factor (VEGF) and negative control proteins (**B**) lysozyme and (**C**) α-chymotrypsin.

The MST binding curves for the VEGF-binding aptamers were obtained, as shown in Figure 1A. The K_D_ value for V7t1-Cy5 was determined to be 201 nM with a standard deviation (SD) of 28 nM. For V7t1-FITC, a K_D_ value of 155 nM with an SD of 55 nM was evaluated. Both K_D_ values of the V7t1 aptamer variants were comparable. Even though different fluorophores were applied to the V7t1 aptamers at different positions, it could be observed that the affinity neither depended on the used fluorophore nor on its position. The lowest K_D_ value of 47 nM was determined for the Del5-1-Cy5 aptamer with an SD of 18 nM.

Moreover, a protein-binding aptamer named Syl3C was used as a negative control. This Syl3C aptamer was selected against EpCAM (epithelial cell adhesion molecule) by Song et al. and contained 48 nucleotides [37], which is a similar length to that of V7t1, which is comprised of 39 nucleotides including the spacer. For this experiment, the EpCAM-binding aptamer Syl3C was labeled with an FITC fluorophore at the 3′-terminus (Syl3C-FITC). The analysis of the negative control aptamer Syl3C incubated with VEGF did not show a binding curve with an amplitude over 5 fluorescence units, as can be seen in Figure 1A. Therefore, it could be concluded that there was hardly any unspecific binding of VEGF to DNA.

The two V7t1 variants and Del5-1 were incubated with negative control proteins lysozyme (Figure 1B) and α-chymotrypsin (Figure 1C). Lysozyme has an isoelectric point (pI) of 11.35, resulting in a positive total charge of lysozyme in TBSET (pH 7) and a molecular weight of 14 kDa. Therefore, it is possible to investigate the unspecific binding between negatively charged DNA and positively charged proteins through electrostatic interactions by using lysozyme as a model protein. The molecular weight of α-chymotrypsin is 25 kDa, which is similar to the molecular weight of the VEGF monomer—23 kDa. Additionally, the pI of α-chymotrypsin, 8.75, is similar to the pI of VEGF, 8.5; therefore, α-chymotrypsin was considered to be a suitable negative control to investigate unspecific binding of VEGF-binding aptamers.

Slightly increased fluorescence intensities were detected for the Del5-1-Cy5, V7t1-FITC and V7t1-Cy5 aptamers at high lysozyme concentrations (Figure 1B). It could be concluded that there is some degree of unspecific binding through electrostatic interactions between the negatively charged VEGF-binding aptamers and positively charged lysozyme at high protein concentrations. Nevertheless, those fluorescence intensities were rather low compared to the fluorescence signals of the binding curves obtained for the three VEGF-binding aptamers. Therefore, it could be assumed that the VEGF-binding aptamers exhibit limited unspecific electrostatic interactions towards positively charged proteins.

In Figure 1C, the fluorescence signals of the VEGF-binding aptamers are shown after incubation with different α-chymotrypsin concentrations. The three aptamers showed a marginal higher fluorescence signal at the highest α-chymotrypsin concentration. It could be concluded that very high protein concentrations (above 3.75 µM) result in a low degree of the unspecific binding of the three VEGF-binding aptamers. Nevertheless, the signal was under 5 fluorescence units, which means hardly any VEGF-binding aptamers bound to the protein with a molecular weight and pI similar to those of VEGF. To ensure that VEGF does not generally bind to DNA, the negative control aptamer Syl3C was incubated with VEGF and investigated by MST, which did not result in the determination of a binding curve.

Even though the K_D_ value of V7t1 was higher than that of Del5-1, V7t1 was used as element for the further development of a TID mechanism, since the 3D-structure of V7t1 contains a G-quadruplex with three G-quartets [13]. The knowledge of the target-binding site of the VEGF-binding aptamer is an advantage for developing a TID assay. Therefore, an oligonucleotide complementary to the G-rich region can be designed [13].

### 3.2. Hybridization and Quenching Effect of Complementary Oligonucleotides

For the development of a TID assay, oligonucleotides complementary to the aptamer sequence are needed. Therefore, an appropriate complementary oligonucleotide has to be designed. Moreover, the hybridization and target-induced dissociation of the oligonucleotides has to be ensured. In this case, a quencher was labeled to the oligonucleotides complementary to the aptamer sequence, and the quenching effect was analyzed via MST using the capillary scan mode. 

**Figure 2:**
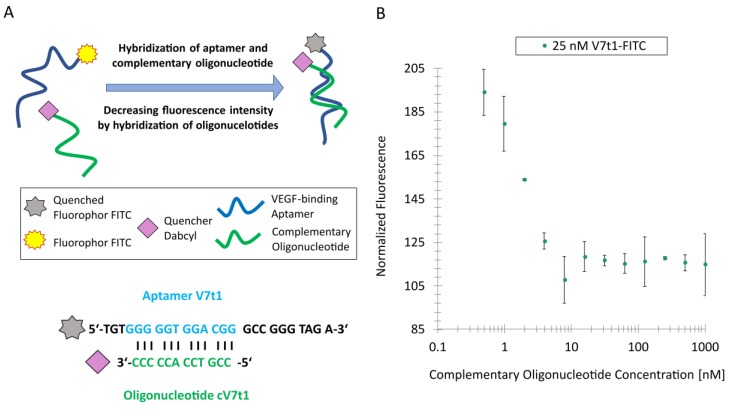
Hybridization of fluorescein isothiocyanate (FITC)-labeled V7t1 aptamer and complementary cV7t1 oligonucleotide modified with a Dabcyl quencher: (**A**) Schematic representation of the oligonucleotide hybridization and quenching effect. The complementary oligonucleotide binds to the G-rich sequence of the V7t1aptamer. (**B**) Normalized fluorescence intensity of the oligonucleotide hybridization and quenching effect depending on different complementary oligonucleotide concentrations.

The G-quadruplex motif of V7t1-FITC was used to design an oligonucleotide complementary to the G-rich region, since the G-quadruplex of this aptamer is known to be important for target-binding. Therefore, the G-quadruplex should be unfolded by hybridizing a complementary oligonucleotide with 12 nucleotides in this region. In presence of the target VEGF, the oligonucleotide complementary to the G-rich region dissociates and the G-quadruplex refolds. For the experiments, the complementary oligonucleotide was modified with a Dabcyl quencher to inhibit the FITC-fluorescence of the aptamer, as schematically seen in Figure 2A.

To ensure the optimal hybridization of the complementary oligonucleotide in the G-rich motif and an optimal quenching effect of the FITC-labeled V7t1 aptamer, the complementary cV7t1 oligonucleotide was designed, as shown in Figure 2A. A distance of three nucleotides between the fluorophore FITC and the Dabcyl quencher guarantees a quenching effect [39]. Moreover, the complementary oligonucleotide length of 12 nucleotides should also ensure the dissociation of the complementary oligonucleotide from the V7t1 aptamer induced by target-binding [31]. An oligonucleotide of the same length has been proven to be optimal in an aptamer-based TID assay for the detection of small molecules when using oligonucleotides binding to the G-quadruplex motif of the aptamer [31].

To determine which concentration of the quencher-modified complementary cV7t1 oligonucleotide is needed to ensure maximal hybridization and thus optimal quenching effect, a constant aptamer concentration of 25 nM of the V7t1-FITC aptamer and different complementary cV7t1-Dabcyl oligonucleotide concentrations in a range of 1 µM to 0.49 nM were used for hybridization. As a result, the maximal fluorescence quenching could already be detected at a complementary oligonucleotide concentration of about 7 nM (Figure 2B). In all accounts, the maximal fluorescence quenching was about 40%. A lower distance from the quencher to the fluorophore might further increase the quenching efficiency of future optimization steps. Another possibility to optimize the assay is the use of a longer oligonucleotide complementary to the aptamer. This could result in an increasing hybridization of the oligonucleotides and could therefore result in a higher quenching efficiency. For the next experiment, a complementary cV7t1 oligonucleotide concentration of 7 nM was used.

### 3.3. TID Analyzed by Capillary Scan

**Figure 3:**
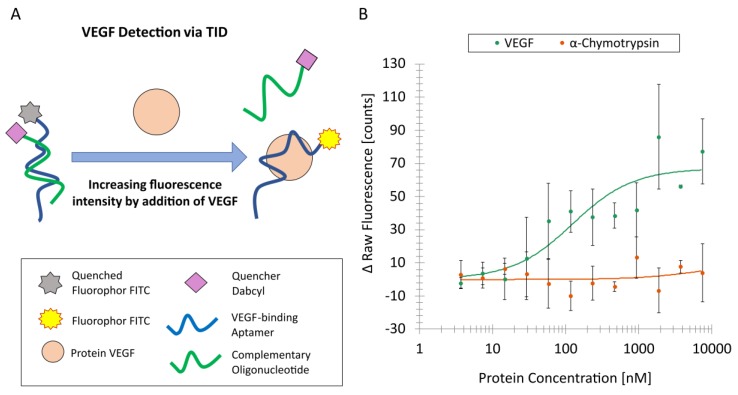
V7t1 aptamer-based detection of VEGF by the target-induced dissociation (TID) of complementary oligonucleotide via microscale thermophoresis (MST) capillary scan. (**A**) Schematic representation of VEGF detection by the TID of complementary oligonucleotide. VEGF replaces the quencher-modified complementary cV7t1-Dabcyl oligonucleotide, which leads to an increasing fluorescence intensity of the FITC-labeled V7t1 aptamer. (**B**) V7t1-FITC aptamer-based VEGF detection by the TID of complementary cV7t1-Dabcyl oligonucleotide via MST capillary scan. The fluorescence intensity depends on the VEGF concentration. Additionally, α-chymotrypsin was used as a negative control.

The schematic presentation of the TID assay which was analyzed using the MST device in the capillary scan mode is shown in Figure 3A.

In the illustrated process, the fluorescently labeled aptamer was hybridized with a quencher-modified complementary oligonucleotide, which resulted in a reduction of the fluorescence intensity. During incubation with the target, the quencher-modified complementary oligonucleotide dissociated from the fluorescently labeled aptamer.

To ensure an optimal hybridization of the V7t1-FITC aptamer to the complementary cV7t1-Dabcyl oligonucleotide, a complementary oligonucleotide concentration of 7 nM was used. The dissociation of the complementary oligonucleotide depended on the used protein concentration, starting at a concentration of 7.5 µM. Again, α-chymotrypsin was used as a negative control. The TID mechanism showed an increasing fluorescence signal with an increasing VEGF concentration by fluorescence analysis (Figure 3B). It could be seen that the complementary oligonucleotide was successfully dissociated by VEGF. The increasing concentration of the negative control α-chymotrypsin barely revealed any effect on the fluorescence intensities (Figure 3B). This leads to the conclusion that the complementary oligonucleotide did not dissociate in presence of α-chymotrypsin. Even though the SD for VEGF detection via the TID assay was high, the assay clearly showed an increasing fluorescence at higher VEGF concentrations in contrast to the negative control. In all accounts, the TID assay for VEGF detection was successful.

### 3.4. TID Assay for VEGF Detection Transferred to Microarrays

After the development of the TID mechanism for VEGF detection via MST and fluorescence analysis, this mechanism was transferred to the microarray format.

**Figure 4:**
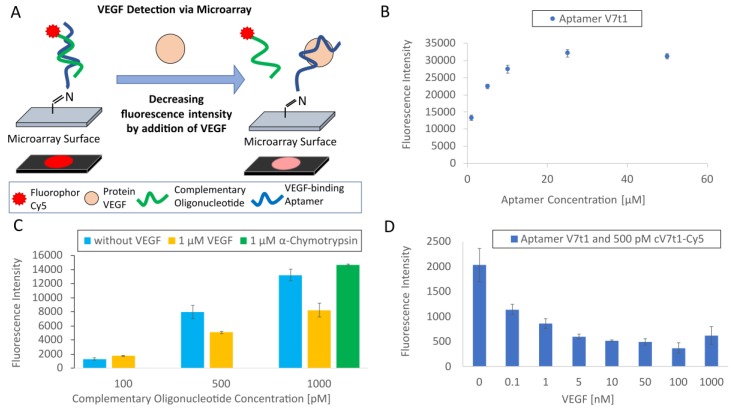
Transfer of the TID assay for VEGF detection to the microarray format. (**A**) Schematic representation of aptamer-based VEGF detection by the target-induced dissociation (TID) of the fluorescently labeled complementary oligonucleotide. The fluorescence signal of the complementary oligonucleotide was reduced by adding VEGF. This image was modified from that of Heilkenbrinker et al. [31]. (**B**) SYBR Green staining of the V7t1 aptamer shows aptamer immobilization onto the microarray surface in dependence of the applied aptamer concentration. (**C**) Oligonucleotide hybridization of V7t1 and cV7t1-Cy5 in dependence of different complementary oligonucleotide concentrations with and without competition with 1 µM of VEGF. Additionally, the negative control protein α-chymotrypsin was used to compete with 1000 pM of complementary oligonucleotide. (**D**) Oligonucleotide hybridization of V7t1 and cV7t1-Cy5 in dependence of different VEGF concentrations for determining the sensitivity of the VEGF detection of the binding assay.

Figure 4A presents an illustration of the microarray-based TID assay for VEGF detection. Thereby, the NH_2_-modified V7t1 aptamer was immobilized to the microarray surface and hybridized with the Cy5-labeled complementary cV7t1 oligonucleotide. The target protein VEGF was added to the oligonucleotides. VEGF could dissociate the complementary oligonucleotide from the aptamer, resulting in decreasing fluorescence intensity.

To confirm the immobilization of the NH_2_-modified V7t1 aptamer on the microarray as well as the binding capacity of the 3D-aldehyde-surface of the microarray, the aptamer was immobilized in different concentrations before starting the TID experiments. The immobilization of the amino-modified V7t1 aptamer on the 3D-aldehyde microarray surface was confirmed using the SYBR Green staining solution, and the binding capacity of the microarray surface was investigated. As a result, the saturation of the aptamer density was achieved at a concentration of 25 µM of aptamer (in the spotting solution), as shown in Figure 4B; this concentration was therefore used for further experiments to hybridize complementary oligonucleotides.

To determine the needed concentration of the Cy5-labeled complementary cV7t1 oligonucleotide, different concentrations were incubated with the V7t1 aptamer immobilized to the microarray surface. Therefore, the incubation step was performed with and without competition with 1 µM of VEGF. The resulting fluorescence intensities are presented in Figure 4C. The incubation of microarrays immobilized with the aptamer (25 µM in the spotting solution) and a concentration of 500 and 1000 pM of Cy5-labeled complementary oligonucleotides (cV7t1) showed a significant lower fluorescence intensity with VEGF than without VEGF. While the use of 100 pM of cV7t1-Cy5 did not reveal any effect, the higher concentrations of 500 and 1000 pM led to a fluorescence decrease of 36% and 38%, respectively, after adding VEGF. Furthermore, no fluorescence decrease could be determined by the use of 1000 pM of cV7t1-Cy5 with the negative control protein α-chymotrypsin (1 µM).

Since 500 pM of the complementary cV7t1-Cy5 oligonucleotide was sufficient to obtain a significant difference in fluorescence intensities between the incubation of the hybridized oligonucleotides with and without VEGF, 500 pM of cV7t1-Cy5 was chosen as a constant concentration in the next experiment. 

The following experiment was accomplished to determine the sensitivity of the TID-based microarray for VEGF detection. Therefore, various VEGF concentrations were incubated with the hybridized oligonucleotides. As shown in Figure 4D, the immobilized V7t1 aptamer incubated with 500 pM of the complementary cV7t1-Cy5 oligonucleotide and different VEGF concentrations revealed a significant lower fluorescence intensity by increasing the VEGF concentration. The dissociation efficiency was 44% at 0.1 nM of VEGF and 70% at 1 µM of VEGF in comparison to the hybridized oligonucleotides without VEGF. It can be assumed that the aptamer-microarray using the TID assay was highly sensitive because it was able to detect 0.1 nM of VEGF.

## 4. Conclusions

In this work, we presented a strategy to systematically develop TID assays by using MST and fluorescence analysis to screen aptamers and complementary sequences for their sustainability and to prove the applicability of the developed system by transferring the TID method onto an aptamer-microarray. 

The MST analysis showed that the VEGF-binding Del5-1 and V7t1 aptamers bind with high affinity and specificity to their target VEGF. The affinity of Del5-1-Cy5 towards VEGF (K_D_ value 47 nM) was even higher than the affinity of V7t1-FITC, with a K_D_ value of 155 nM. Nevertheless, V7t1 contains a G-quadruplex which is known to be important for the binding of VEGF and can be used for the development of a TID mechanism; however, the binding site of Del5-1 has yet to be revealed. Nonetheless, the proposed strategy is also applicable for the investigation of the aptamer binding site by screening different complementary oligonucleotides against a given aptamer, thereby identifying oligonucleotides that can be dissociated due to aptamer–target interaction.

In case of V7t1, a quencher-modified oligonucleotide complementary to the G-quadruplex motif of V7t1 was designed and hybridized to the V7t1-FITC aptamer. The maximal fluorescence quenching was obtained at a complementary oligonucleotide concentration of 7 nM. Afterwards, the TID mechanism was investigated by adding VEGF in different concentrations to the aptamer–oligonucleotide complex, thus revealing an increasing fluorescence signal with increasing VEGF concentration.

Finally, the method was transferred to an aptamer-microarray to prove the applicability of the developed TID assay. The incubation of microarrays with 500 pM of Cy5-labeled complementary oligonucleotides (cV7t1) competing with VEGF showed a significant lower fluorescence intensity than without VEGF. This leads to the conclusion that 500 pM of cV7t1-Cy5 was an appropriate concentration for hybridizing with the aptamer. Nonetheless, the concentration of the complementary oligonucleotide could be further optimized. Here, it has to be carefully considered that an increase of the oligonucleotide concentration will not only result in higher signal intensities, but the excess of oligonucleotide will also compete with VEGF for binding to the aptamer, which could impair the microarray sensitivity. At last, the sensitivity of the VEGF detection via TID mechanism on the developed aptamer-microarray was determined. Therefore, the immobilized aptamer (25 µM in the spotting solution) was incubated with its complementary oligonucleotide (500 pM) before adding VEGF. The difference between the fluorescence intensity at 0.1 nM of VEGF was 44% in contrast to the hybridized oligonucleotides without VEGF, suggesting that even lower VEGF concentrations might be detectable. Therefore, the sensitivity of the microarray could also be investigated with lower VEGF concentrations in future experiments.

An advantage of the TID assay is its high specificity, since not every binding results in a signal. In this study, an aptamer-based TID assay was schematically developed by MST and fluorescence analysis for the detection of the VEGF model protein. Furthermore, the TID assay was successfully transferred to a different application—an aptamer-microarray. All in all, we propose that the strategy to develop TID assays can be transferred to further aptamers, and the developed VEGF-sensitive TID assay could be transferred to different biosensing platforms for medical research and diagnostics in the future.

## Figures and Tables

**Table 1:** Oligonucleotide sequences with type and position of modification, their corresponding target and manufacturer.

Oligonucleotides	Sequence (5′ → 3′)	Modification	Manufacturer	Target
cV7t1-Cy5	CCGTCCACCCCC	3′-terminal Cy5	Integrated DNA Technologies, Coralville, IA, USA	V7t1
cV7t1-Dabcyl	CCGTCCACCCCC	3′-terminal Dabcyl	Integrated DNA Technologies, Coralville, IA, USA	V7t1
V7t1-FITC	TGTGGGGGTGGACGGGCCGGGTAGATAGTATGTGCATTC	5′-terminal FITC and 3′-terminal 14 nt spacer	Integrated DNA Technologies, Coralville, IA, USA	VEGF
V7t1-NH_2_	TGTGGGGGTGGACGGGCCGGGTAGATAGTATGTGCATTC	14 nt spacer with 3′-terminal NH_2_	Integrated DNA Technologies, Coralville, IA, USA	VEGF
V7t1-Cy5	TGTGGGGGTGGACGGGCCGGGTAGATAGTATGTGCAATC	14 nt spacer with 3′-terminal Cy5	Biomers, Ulm, Germany	VEGF
Del5-1-Cy5	ATACCAGTCTATTCAATTGGGCCCGTCCGTATGGTGGGTGTGCTGGCCAG	3′-Cy5	Biomers, Ulm Germany	VEGF
Syl3C-FITC	CACTACAGAGGTTGCGTCTGTCCCACGTTGTCATGGGGGGTTGGCCTG	3′-FITC	Biomers, Ulm Germany	EpCAM

Grey highlighted sequence: 14 nt spacer sequence.
